# Saprotrophic Capabilities of *Neurospora crassa* on Charred Plant Biomass

**DOI:** 10.1111/1462-2920.70132

**Published:** 2025-06-24

**Authors:** Hunter J. Simpson, Jonathan S. Schilling

**Affiliations:** ^1^ Department of Bioproducts and Biosystems Engineering University of Minnesota St. Paul Minnesota USA; ^2^ Plant and Microbial Biology University of Minnesota St. Paul Minnesota USA

**Keywords:** fire adaptation, fire ecology, fungal ecology, grass decay, lignocellulose, PyOM, wood decay

## Abstract

*Neurospora crassa* is a popular model organism for laboratory research, yet its natural ecology remains mysterious. Its proliferation on charred plant biomass (wood and grasses) in fire‐affected environments is often linked to the heat tolerance or heat−/chemical‐induced germination of *N. crassa* spores; however, this link is not consistent across ecosystems or substrate types. Another possible, yet unvalidated, explanation is that *N. crassa* has an enhanced capacity for degrading charred (i.e., pyrolyzed) plant biomass. We assessed this adaptation for *N. crassa* by quantifying the decay of wood and grasses that were pyrolyzed to relevant extents (untreated, heated at 225°C or 350°C for 20 min) and by comparing this decay with non‐fire‐associated fungi. *Neurospora crassa* did not have an enhanced ability to degrade pyrolyzed substrates. Additionally, *N. crassa* struggled to degrade any wood substrate (< 6% mass loss) but did degrade untreated grasses (> 20% mass loss). These results, paired with chemical analyses of substrates pre‐ and post‐decay, support a fire‐response strategy for *N. crassa* , rather than a fire‐adaptive ability to degrade charred substrates. This fungus likely proliferates on charred biomass by rapidly colonising heat‐sterilised substrates after heat‐ or smoke‐induced spore germination and then consuming unpyrolyzed lignocellulose beneath a charred exterior.

## Graphical abstract

Despite growing on charred biomass in nature, *Neurospora crassa* cannot degrade charred wood or grass better than non‐fire‐associated decomposer fungi (*Gloeophyllum trabeum, Trametes versicolor*). It likely proliferates on charred biomass by rapidly colonising heat‐sterilised substrates after heat‐ or smoke‐induced spore germination and then consuming unpyrolyzed lignocellulose beneath a charred exterior.

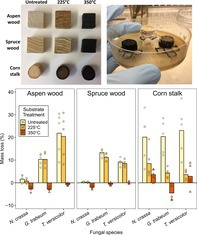

## 1 Introduction

Despite its use as a model organism for nearly 100 years (Roche et al. 2014) and its facilitation of paradigm‐shifting research (e.g., validation of the ‘one gene, one enzyme’ hypothesis; Beadle and Tatum 1941), the ecology of *Neurospora crassa* in nature has remained largely enigmatic. We often associate *Neurospora* species with plant substrates used in industry (e.g., bread, cooked corn cobs, sugarcane refinery filter mud, steamed soil and sawdust; Shear and Dodge 1927; Perkins et al. 1976; Perkins and Turner 1988; Turner et al. 2001), but in nature, the conidiating species of *Neurospora*, including *N. crassa* , are most often found growing on burned (i.e., charred or pyrolyzed; not fully combusted) plant material post‐fire. These charred substrates include trees (e.g., *Pinus* spp., *Populus* spp.; Jacobson et al. 2004), as well as grasses (e.g., sugarcane; Perkins et al. 1976; Powell et al. 2003; Jacobson et al. 2006).

To explain why and how *Neurospora* is associated with burned plant material, many have implicated the heat tolerance or heat requirement of *Neurospora* spores. For example, desiccated conidiospores of *N. crassa* have been shown to survive temperatures greater than 100°C for 15–20 min (Shear and Dodge 1927). Ascospores have demonstrated heat tolerance as well, surviving temperatures up to 67°C for ~200 min (Lindegren 1932). Both types of heat‐tolerant spores could survive the heat of a fire and germinate on or near freshly killed and heat‐sterilised plant biomass with little competition from other saprotrophs. This would represent a combined ecological strategy of fire‐resistance and fire‐responsiveness, as defined by Hopkins and Bennett (2024). *Neurospora* ascospores have also been suggested to *require* heat (Shear and Dodge 1927; Lindegren 1932) or fire‐derived chemicals (e.g., furfural; Emerson 1948; Emerson 1954; Eilers and Sussman 1970; Pandit and Maheshwari 1996) to germinate. This represents a similar ecological strategy and further supports a more *ruderal* fire‐response, as induced germination would enable more rapid proliferation in a post‐fire environment. Pandit and Maheshwari (1996) confirmed this strategy in burned sugar cane fields by demonstrating that *Neurospora* ascospores in soil were activated by fire‐derived furfural, and that the emerging hyphae then colonised fire‐killed sugar cane stumps. Studies of *Neurospora* in other fire‐affected environments, however, have been unable to confidently identify the same mechanisms for colonisation (e.g., Jacobson et al. 2004; Kuo et al. 2014), leaving doubt about why fire‐affected substrates are a niche for *Neurospora*.

An alternative, or perhaps complementary, explanation for fire association is that *Neurospora* may have enhanced metabolic capacities to degrade charred plant material. This type of pyrogenic organic matter (PyOM) metabolism is defined as a ‘fire‐adaptive’ trait by Hopkins and Bennett (2024). When plant matter, especially material with smaller surface area to volume ratios (e.g., trees, logs, branches, and large stems, and stalks), burns during a wild or prescribed fire, it often does so incompletely (i.e., is not completely combusted into ash), leaving behind a mosaic of partially burned lignocellulose residues that have varying degrees of chemical modification. These chemical modifications are the result of pyrolysis, which, after all water is vaporised from the substrate, is the first step in biomass combustion.

Pyrolysis of plant biomass involves the thermal degradation (at temperatures between 225°C and 500°C; Tillman et al. 1981) and volatilization of lignocellulose polymers. These volatile degradation products then escape the biomass, mix with air, and combust to produce a flame, which produces heat that fuels further pyrolysis within the substrate (i.e., self‐sustained combustion). However, as mentioned above, the conditions for complete pyrolysis and combustion of plant biomass are not always sustained during a fire. Depending on the temperature and duration of heat exposure, much of the polysaccharides (cellulose, hemicellulose) in the lignocellulose may be lost (i.e., volatilized), while much of the lignin may be converted into char residues (Tillman et al. 1981), which are notoriously recalcitrant to degradation (Czimczik and Masiello 2007). Additionally, some of the degradation products of both polysaccharides and lignin (e.g., furans and phenolics; Highley 1975, Modig et al. 2002), which may still be present in the substrate, may inhibit microbial growth. The ability to overcome these decay challenges would undoubtedly provide a competitive advantage in an environment where these residues are plentiful. While there is some qualitative evidence to suggest that some fire‐associated fungi may have an enhanced ability to grow on charred substrates (Edman and Eriksson 2016; Fischer et al. 2021), this has not been tested for *Neurospora* species. Additionally, there have been no quantitative decay studies (i.e., quantified mass loss from charred substrates) to confirm this adaptation for any fungus, to the best of our knowledge.

In this study, we sought to answer if *Neurospora* has enhanced capacities to degrade charred substrates, relative to non‐fire‐associated fungi tested within the same treatment structure. We specifically used single‐strain microcosms to assess the capacity of *Neurospora crassa* to degrade grass and woody substrates heat‐treated at field‐relevant temperatures, comparing mass loss in heat‐treated substrates to non‐treated substrates. We also compared mass loss produced by *N. crassa* with mass loss produced in the same substrate treatments by two non‐fire associated fungi, a ‘white rot’ lignin‐degrading fungus (*Trametes versicolor*), and a ‘brown rot’ carbohydrate‐selective fungus (*Gloeophyllum trabeum*). In addition to mass loss, we tracked losses of lignin and structural carbohydrates to gain insight into the biochemical decay pathways of *N. crassa* . Our overarching logic was that by linking ecology of fire‐association to underlying biological traits in *N. crassa* , one of the most tractable genetic/genomic fungal resource among all life forms (Galagan et al. 2003), we might highlight fruitful avenues to deepen valuable trait‐function relationships.

## 2 Experimental Procedures

This study included an agar‐block microcosm trial to compare *N. crassa* with wood‐decaying fungi using solid, non‐milled substrates. The study also included two soil‐based microcosm trials, one trial as a time series using solid corn stalk discs and a second trial using milled corn stalks presented in mesh ‘litter bags’.

### 2.1 Substrates

For the solid substrates used in the agar‐block experiment, quaking aspen ( *Populus tremuloides* ) sapwood, white spruce ( *Picea glauca* ) sapwood, and corn ( *Zea mays* ) stalk internodes were cut to uniform dimensions. Wood substrates were cut into 19 × 19 × 9.5 mm blocks, whereas corn stalks were cut into disks with a thickness of 9.5 mm and a diameter of roughly 15 mm. All substrates were subjected to three different temperature treatments, including no treatment (i.e., untreated), heated at 225°C, and heated at 350°C. These temperatures were chosen because hemicellulose begins to decompose at 225°C, and most hemicellulose and some cellulose are decomposed at 350°C (Rowell and Dietenberger 2012); thus, these temperatures were suitable targets for creating a reasonable spectrum of severity in thermal modification. Heat treatments were performed in a muffle furnace by first placing the blocks or disks within covered Pyrex petri dishes (100 × 20 mm) and then adding these dishes to the preheated furnace for 20 min. Dishes remained covered to reduce oxygen exposure and possible combustion (i.e., substrates were pyrolyzed). After testing various durations to ensure even heating throughout the entirety of each substrate, a treatment time of 20 min was deemed suitable, as judged by a consistent colour change from the exterior to the interior of each substrate. After treatment, dishes were allowed to cool to room temperature before removing the lids. For a visual comparison of heat‐treated substrates, see Figure S1. To quantitatively judge the severity of each heat treatment, mass loss was quantified by measuring oven‐dried (i.e., dried at 100°C for 48 h) mass before and after heat treatment for a three‐replicate subset (i.e., three replicates per treatment temperature) of each substrate. The mass loss for aspen, spruce, and corn stalk at 225°C was 0.5% (±0.2 SD), 0.3% (±0.1 SD), and 10.7% (±2.7 SD), respectively, whereas the mass loss at 350°C was 62.0% (±1.8 SD), 57.9% (±2.8 SD), and 56.4% (±1.8 SD), respectively (Figure S2). After heat treatment, the oven‐dried masses of all substrates were recorded, and then all substrates were autoclaved for 1 h (121°C, 103 kPa). Once cooled, sterilised substrates were added to each microcosm. When substrates were removed at the end of the experiment, superficial hyphae were gently rubbed off, and the oven‐dried mass of each substrate was recorded to calculate the mass loss during decay. Note that all decay mass loss values we report have been adjusted by subtracting mass loss (due to leaching) of non‐inoculated controls.

For the time series experiment in soil‐based microcosms, corn stalk internodes with a slightly larger diameter of 20 mm were cut uniformly to 9.5 mm thickness. Once cut, corn stalk disks were treated as outlined above, but with three substrates in each microcosm removed at different time points (i.e., 10, 20, 40 days of decay) as opposed to one harvest time. Removed substrates were cleaned of superficial hyphae and then oven‐dried to calculate mass loss.

For the soil‐based microcosm experiment, milled stalks of corn, sorghum ( *Sorghum bicolor* ), and wheat ( *Triticum aestivum* ) were used as grass substrates. Sorghum and wheat were chosen for comparison with corn because they represent closely (i.e., within the same subfamily, Panicoideae) and distantly (i.e., within a different subfamily, Pooideae) related grass species, respectively. The internodes of sorghum and corn stalks and a mixture of wheat stems and leaves (i.e., wheat straw; small stem size made isolation from leaves impractical) were milled through 20 mesh (841 μm) using a Wiley mill. To test for decay differences due to a differing microcosm setup (i.e., powders in mesh bags versus whole blocks or disks), heated corn stalk substrates (i.e., heated at 225°C or 350°C) and aspen were, again, included as substrates. Heat‐treated corn stalk powder was produced using similar techniques as outlined above, except Pyrex dishes were filled with powder instead of whole corn stalk disks. For aspen substrates, sapwood was ground to 20 mesh and left untreated. To test for the effect of water‐soluble compounds (e.g., sucrose, glucose, fructose) on the overall mass loss from corn stalks, corn stalk powder that was pre‐extracted with distilled water was also included as a substrate. Powder was water‐extracted in bulk by adding ~80 g of untreated corn stalk powder to a Buchner funnel with filter paper and slowly pouring 16 L of distilled water through the powder. After preparing all substrates, powders were added to 6 × 6 cm nylon mesh bags with a pore size of 52 μm. Oven‐dried mass (i.e., dried at 100°C for 24 h) of empty bags was recorded before filling them with substrates. Filled bags were also oven‐dried (i.e., 100°C; 24 h), then weighed and autoclaved for 1 h (121°C, 103 kPa). Once cooled, sterilised bags were added to each microcosm. When bags were removed at the end of the experiment, superficial hyphae were gently rubbed off and the oven‐dried mass of each bag was recorded to calculate mass loss during decay.

### 2.2 Microcosms

To compare the decay abilities of *Neurospora crassa* with non‐fire‐associated fungi, deep dish (100 × 25 mm) agar‐block microcosms were used to quantify and compare mass loss from various lignocellulose substrates. Microcosms were prepared with potato dextrose agar (PDA), using methods similar to those used by Schilling and Jacobson (2011), and then inoculated with *Neurospora crassa* 74‐OR23‐1VA (FGSC 2489), *Gloeophyllum trabeum* ATCC 11539, or *Trametes versicolor* A1‐ATF (Forest Pathology Culture Collection, University of Minnesota, USA) using 1‐cm diameter agar plugs from 1 to 2 week‐old PDA inoculum cultures. *G. trabeum* and *T. versicolor* were chosen for comparison with *N. crassa* because they have established use in decay comparison studies (ASTM 2007), have well‐documented wood and grass decay abilities (Kaffenberger and Schilling 2013, 2015; Osono 2010), utilise two different lignocellulose decay strategies (i.e., brown vs. white rot decay, respectively), and have no clear association with burned environments. Substrates (i.e., aspen blocks, spruce blocks, corn stalk disks) were simultaneously added to each culture by placing one of each substrate treatment (i.e., untreated, 225°C, 350°C; see Figure S1) on a single plate. This experimental layout resulted in a total of 60 plates when accounting for five replicates and non‐inoculated controls. When adding substrates, they were first submerged in sterile distilled water for 5–10 s then placed on sterilised high‐density polyethylene screens (Amerimax Home Products, Lancaster, PA, USA). The screens were added to each culture to reduce anoxia due to moisture wicking from agar into the substrates. For more details on the agar‐block microcosm layout, see Figure 2. Parafilmed microcosms were maintained at room temperature (~25°C), without direct humidity control, for 35 days.

Soil‐jar microcosms were used for analysing *N. crassa* decay abilities by studying corn stalk decay over time and decay of various grass species, in comparison to corn. These microcosms more closely resemble natural decay conditions, in comparison to agar‐block microcosms. Microcosms were prepared based on the method outlined by ASTM (2007). To summarise, pint glass jars were filled one‐third full with a 1:1:1 mixture of soil (fertiliser‐free), peat, and vermiculite hydrated to roughly 35% (wt vol^−1^) moisture content. Two birch feeder strips (40 × 10 × 2 mm) were placed on top of the soil in each jar, and the jars were then autoclaved twice for 1 h (121°C, 103 kPa) with a 24‐h interval between the two cycles. Jars were allowed to cool for 24 h before being inoculated with *N. crassa* , using four 1‐cm diameter agar plugs from one‐week‐old PDA inoculum cultures. For the time series experiment, cultures were grown for 2 weeks to allow for colonisation of the feeder strips before adding the corn stalk substrates to the mycelial mat. Three substrates of the same treatment were added to each jar to account for three different harvest points (10, 20, 40 days of decay). This resulted in a total of 30 jars when accounting for five replicate jars and non‐inoculated controls. To test decay of various grass species, jars were prepared in a similar fashion, but substrates were added to each jar as powders contained within mesh bags. In the first two experiments, whole (non‐powder) substrates were used, which resulted in a considerable amount of variability in the quantified mass loss values for corn stalk discs. This may have been due to variability in the amount of water‐soluble compounds between each disc, which could have led to substantial variability in mass loss due to leaching or decay (i.e., water‐soluble compounds, like sucrose, are easier to degrade than lignocellulose). To reduce this variability in our third experiment, we chose to homogenise each substrate into a stock powder that was then aliquoted into mesh bags. Only one bag was added to each jar, which resulted in 70 jars when accounting for the seven substrates tested (i.e., sorghum stalk, wheat stalk, untreated corn stalk, corn stalk heated at 225°C, corn stalk heated at 350°C, corn stalk pre‐extracted with water, aspen), five replicates, and non‐inoculated controls. For both soil‐jar microcosm experiments, substrates were submerged in sterile distilled water for 5–10 s before being added atop the mycelial mat in each jar. For more details on the soil‐jar microcosm layouts see Figures S3 and S4. Microcosms for the time series and grass decay experiments were maintained at room temperature (~25°C), without direct humidity control, for 40 and 35 days, respectively.

### 2.3 Chemical Characterisation

Non‐heat‐treated corn stalks from the grass decay experiment were chemically characterised after decay by *N. crassa* to elucidate decay patterns and compare the ability of *N. crassa* to degrade water‐soluble versus lignocellulose compounds. To quantify water‐soluble compounds, 0.25 g of oven‐dried (i.e., dried at 100°C for 24 h) decayed and undecayed (i.e., control) corn stalk powders (20 mesh) were placed in 125 mL Erlenmeyer flasks along with 25 mL of distilled water. Flasks were then shaken at 200 RPM for 24 h at 4°C. After extraction, flask contents were filtered through glass Gooch crucibles (~5 μm filter pores), which were previously oven‐dried and weighed. Filtrates were collected and stored at −20°C, while crucibles and retained solids were oven‐dried and weighed. The mass of total water‐soluble compounds (i.e., ‘total solubles’) was determined by comparing substrate mass before and after extraction (i.e., crucible mass subtracted from total mass), with the remaining non‐solubilised substrate mass corresponding to ‘total insolubles’. The mass of total solubles lost during decay was determined by comparing total solubles content between the decayed and non‐inoculated control samples. The same was done for mass loss of total insolubles. Degradation of total solubles was further assessed by quantifying specific water‐soluble compounds in the decayed and control samples. Sucrose, glucose, and fructose within the water filtrates were quantified using high performance liquid chromatography (HPLC; Agilent 1200 series; Agilent USA, Santa Clara, CA). Specifically, compounds were measured with a refraction index detector (RID) after being separated by an Aminex HPX‐87P column (Bio‐Rad Laboratories Inc., Hercules, CA, USA; model #1250098) equipped with an in‐line de‐ashing guard column (Bio‐Rad; model #1250118). A flow rate of 0.6 mL min^−1^ was used for the mobile phase (HPLC‐grade water), and the column heater was maintained at 85°C. The masses for each sugar were quantified in decayed and control filtrates and then used to determine the mass of each component lost during decay. Note that some of the total solubles mass was unaccounted for after quantifying sucrose, glucose, and fructose mass and this unknown fraction is reported as ‘other solubles’.

Degradation of total insolubles (e.g., lignin, polysaccharides) was further assessed using a modified National Renewable Energy Laboratory (NREL) procedure (Sluiter et al. 2008) outlined by Zurawski et al. (2015). In brief, lignocellulose samples were treated with sulfuric acid, which hydrolyzes polysaccharides into soluble monomeric sugars but leaves much of the lignin intact. After hydrolyzing our water‐extracted corn stalk samples, hydrolysates were filtered through G6‐grade glass fibre filters (Thermo Fisher Scientific Inc., Waltham, MA, USA; catalogue #09‐804‐55A) in a Buchner funnel. Filters were oven‐dried and weighed prior to use. Filtrates were collected and stored at 4°C, while filters and retained solids were oven‐dried and weighed. To determine the amount of ash in these retained solids, filters were treated in a muffle furnace using the methods outlined in the NREL protocol. No ash was detected, which is reasonable when considering previous analyses of acid‐insoluble ash (i.e., silica) in corn stalks (Johnson et al. 2007; Li et al. 2012); thus, all acid‐insoluble material was assumed to be acid‐insoluble lignin (AIL). Due to notable issues (Sluiter et al. 2010) with the NREL method for acid‐soluble lignin (ASL) quantification, we did not calculate ASL and therefore refer to AIL simply as ‘lignin’. While we did attempt to calculate acid‐insoluble ash, we chose not to calculate total ash, which includes water‐soluble and acid‐soluble ash, due to our lack of interest in ash ‘degradation’ by *N. crassa* ; however, this component is a likely constituent of the unknown fractions for lignocellulose (i.e., ‘other insolubles’) and water‐soluble compounds (i.e., ‘other solubles’), which are discussed in more detail below.

Polysaccharide‐derived compounds in the filtrate were quantified using methods similar to those used for water‐soluble compounds. After neutralising the acidic filtrates with calcium carbonate, glucose, xylose, and arabinose, which are the primary sugars derived from grass lignocellulose, were all separated with the Aminex HPX‐87P and measured using an RID. Hydroxymethylfurfural (HMF) and furfural were also separated with the 87P but were measured with a multiple wavelength detector (MWD) using a wavelength of 280 nm. These two compounds are produced in minor quantities during acid hydrolysis due to the thermal degradation of six‐ and five‐carbon sugars, respectively. While minor, quantifying these compounds can prove useful in helping close carbon balances during chemical characterisation (Whitfield et al. 2016). Acetic acid, produced when acetate is removed from hemicellulose during acid hydrolysis, was separated using an Aminex‐HPX‐87H column (Bio‐Rad; model #1250140) equipped with a Micro‐Guard Cation H guard column (Bio‐Rad; model #1250129) and then measured with an RID. A flow rate of 0.6 mL min^−1^ was used for the mobile phase (5 mM sulfuric acid), and the column heater was maintained at 65°C. The masses of polysaccharide constituents, pre‐acid hydrolysis (i.e., glucan, xylan, arabinan, acetate), were calculated via stoichiometric conversion, with the masses of glucose and HMF being used for glucan calculation, xylose for xylan, arabinose for arabinan, and acetic acid for acetate. Because furfural can be derived from any five‐carbon sugar, it was impossible to assign it to either xylan or arabinan. Consequently, we report the polysaccharide equivalent of furfural generically as ‘C5‐sugar’. Once quantified, masses for each lignocellulose component in decayed and control substrates were used to determine the mass loss during decay. Note that some of the total insolubles mass was unaccounted for after quantifying the mass of these specific components and this unknown fraction is reported as ‘other solubles’.

### 2.4 Statistical Analyses

All statistical testing and figure creation was done in R (R Core Team 2020). Figures were generated using the ggplot2 package (Wickham 2016), while statistical tests were done using rstatix (Kassambara 2021) and multcomp (Hothorn et al. 2008). When normality and homoscedasticity assumptions were satisfied (normality verified with Shapiro–Wilk tests and density plots; homoscedasticity verified with Levene's test), selected groups were compared using t‐tests (rstatix::pairwise_*t*_test) and Bonferroni‐Holm corrections to account for multiple comparisons. In the cases where data were not normal, Mann–Whitney *U* tests (rstatix::pairwise_wilcox_test) were used for comparing selected groups. For the grass decay experiment specifically, all groups were compared via ANOVA and the post hoc Tukey HSD test (multcomp::glht). For comparing corn stalk chemical components, paired *t*‐tests were used to account for dependent samples. Differences between groups were judged significant when *p* values were less than 0.05.

## 3 Results

### 3.1 Decay of Heat‐Treated Substrates—*Neurospora Crassa* Versus Non‐Fire‐Associated Species

**FIGURE 1:**
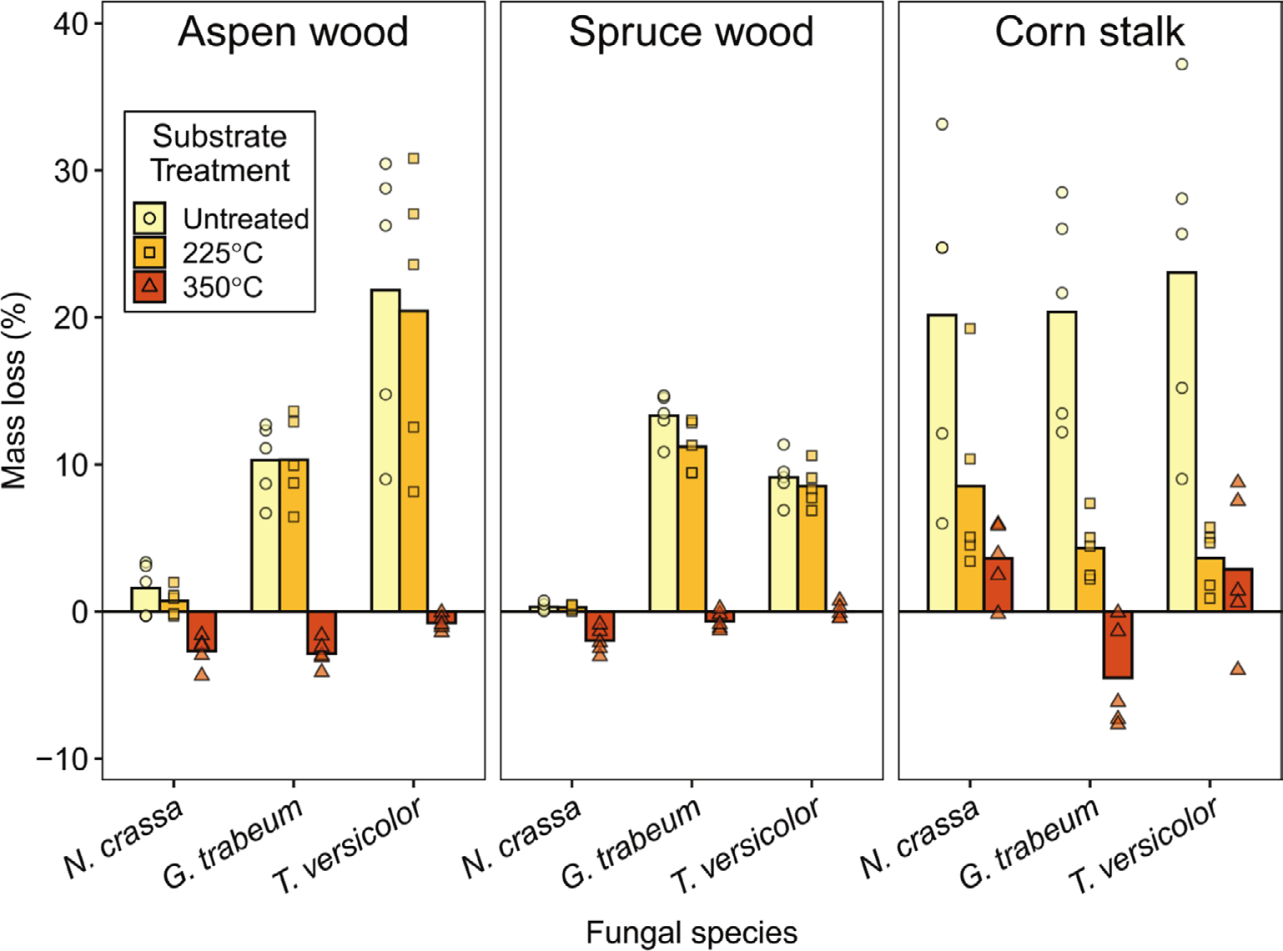
Mass loss of lignocellulose substrates in agar plates after 5 weeks of decay by *Neurospora crassa*, *Trametes versicolor*, or *Gloeophyllum trabeum*. Bars and points indicate mass loss averages and mass loss of individual replicates (*n* = 5), respectively. Substrates, pre‐decay, were untreated, heated at 225°C for 20 min, or heated at 350°C for 20 min.

*Neurospora crassa* did not degrade any of the heat‐treated substrates better than *Gloeophyllum trabeum* or *Trametes versicolor* (Figure 1; *p* < 0.05; as judged by separate multiple comparison tests for each substrate type). This was the case for untreated substrates as well, except for corn stalk (mass loss was equivalent between the three fungal species; *p* < 0.05). When comparing the degradation of different untreated substrates for *N. crassa* only, corn stalk degradation (20.1% (±10.9 SD)) was significantly greater than that for either wood substrate (*p* < 0.05), which were both close to zero (aspen: 1.6% (±1.8 SD); spruce: 0.3% (±0.3 SD)). Finally, it is notable that mass loss generally decreased with increasing treatment severity based on aggregated mean mass loss values (i.e., aggregated across fungal species and substrate type) of 13.3% (±10.2 SD), 7.6% (±7.1 SD), and −0.8% (±3.3 SD) for all untreated, 225°C‐treated, and 350°C‐treated substrates, respectively. Note that negative mass loss values likely indicate colonisation by fungal biomass (mass gain) without any substantial substrate metabolism (mass loss).

**FIGURE 2:**
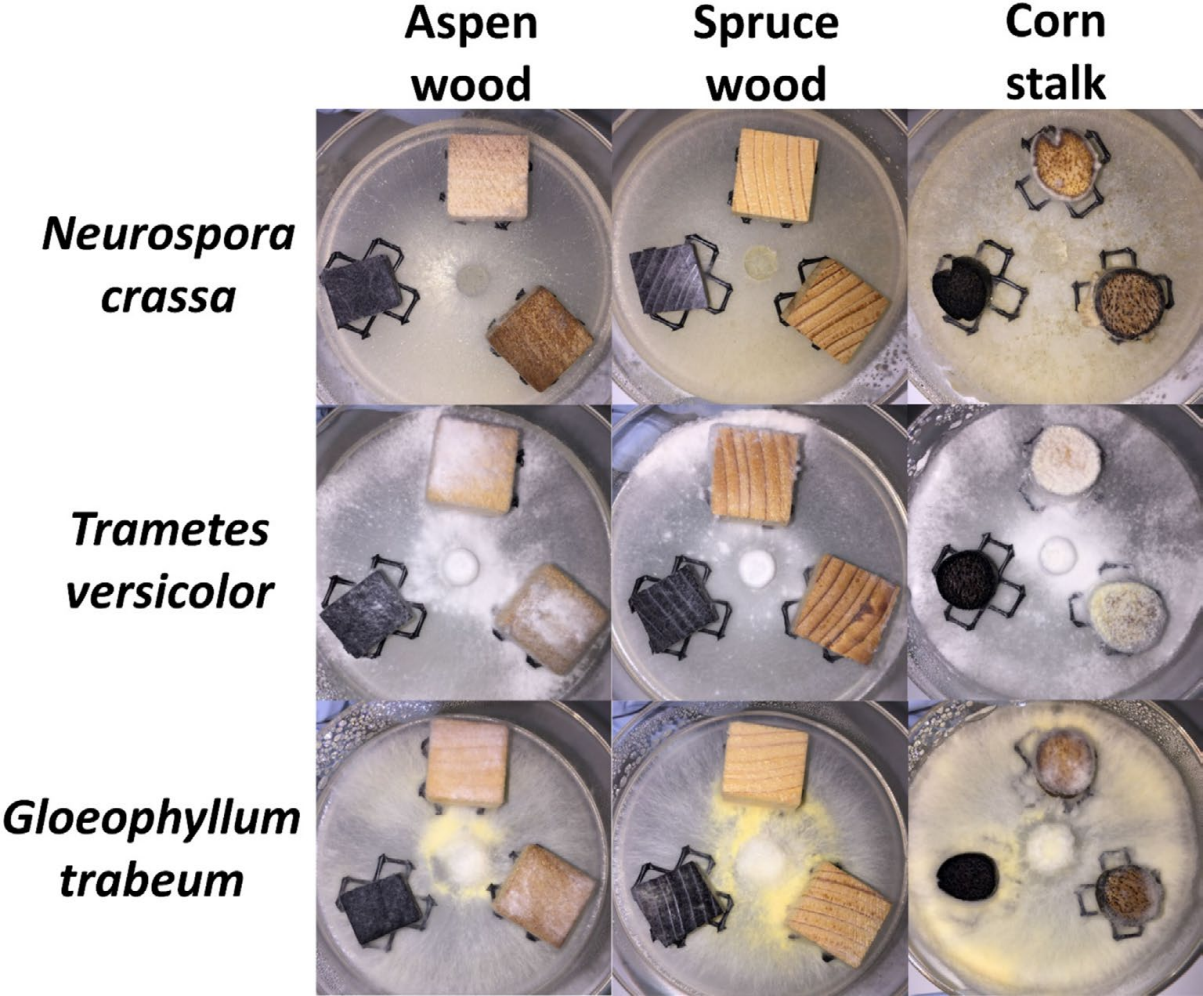
Agar‐block microcosms after 11 days of growth by *Neurospora crassa*, *Trametes versicolor*, and *Gloeophyllum trabeum*. Substrates include aspen and spruce sapwood blocks, as well as corn stalk internode disks. Substrate treatments, all of which can be seen in the same microcosm, include untreated, heated at 225°C for 20 min, or heated at 350°C for 20 min. Substrates placed on high‐density polyethylene screens to reduce moisture wicking from PDA medium.

Methods for estimating fungal biomass (e.g., ergosterol, chitin, qPCR) within a solid substrate each have their own caveats (Song et al. 2014) and ultimately fail to provide a robust solution for quantifying mass loss without the interference of fungal biomass addition in comparative decay analyses; therefore, we did not attempt to estimate fungal biomass (which is common for comparative decay analyses; e.g., Kaffenberger and Schilling 2015) but did visually assess the cultures (Figure 2) to ensure that mass loss values were not heavily skewed due to stark differences in substrate colonisation. For example, *N. crassa* is known for prolific growth, and it is possible that mass loss values may be skewed low due to the large addition of fungal biomass to the substrate; however, Figure 2 shows that *N. crassa* was not noticeably more aggressive than the other two species and the possibility that we are underestimating mass loss by *N. crassa* relative to the other two species seems unlikely. It is also important to note that fungal biomass addition likely had a larger impact on calculated mass loss for the substrates treated at 350°C due to their increased porosity and lower starting mass (see Figure S2), compared to the other substrates. Fungal hyphae were likely able to penetrate the substrate easier (and these hyphae could not be easily removed before calculating mass loss, as was done for superficial hyphae), and the ratio of fungal biomass to substrate mass was likely greater.

### 3.2 Decay of Heat‐Treated Substrates Over Time by *Neurospora Crassa*

**FIGURE 3:**
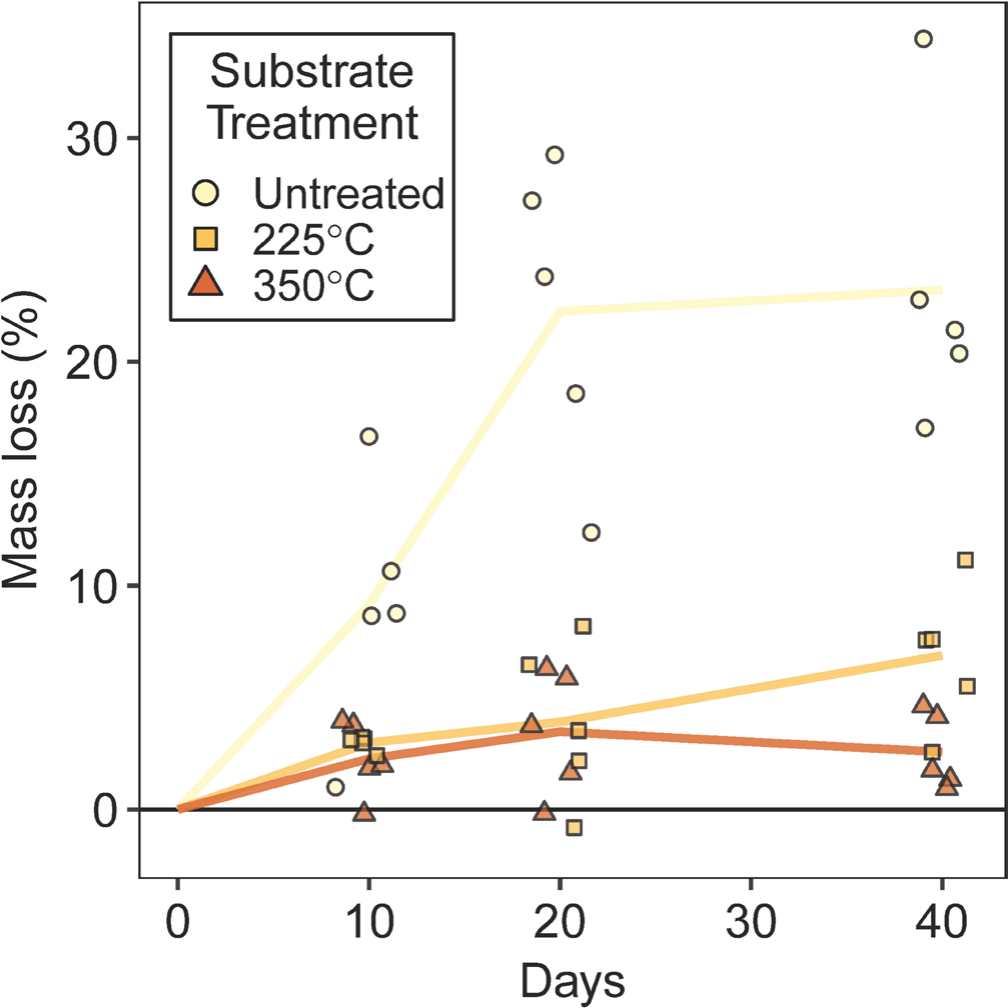
Mass loss of corn stalk discs in soil‐filled jars after 0, 10, 20, or 40 days of decay by *Neurospora crassa*. Lines connect mass loss averages at each time point, whereas points indicate mass loss for individual replicates (*n* = 5). Corn stalk discs, pre‐decay, were untreated, heated at 225°C for 20 min, or heated at 350°C for 20 min. *X*‐axis jitter added to data at each time point to enhance point visibility.

Temporal patterns of corn stalk mass loss during decay by *N. crassa* varied with heat‐treatment, but values for all treatments reached a plateau within the 40‐day growth period (Figure 3). For the 225°C‐treated and 350°C‐treated substrates, mass loss at 40 days was 6.9% (±3.2 SD) and 2.6% (±1.7 SD), respectively, and these values were not significantly different than those obtained at 10 days (*p* < 0.05). Conversely, mass loss for untreated corn stalk at 40 days was 23.2% (±6.6 SD) and was significantly greater than the corresponding value at 10 days (9.2% (±5.6 SD); *p* < 0.05); however, there was no significant difference in mass loss between 20 and 40 days (*p* < 0.05).

### 3.3 Decay of Various Grass Species by *Neurospora Crassa*

**FIGURE 4:**
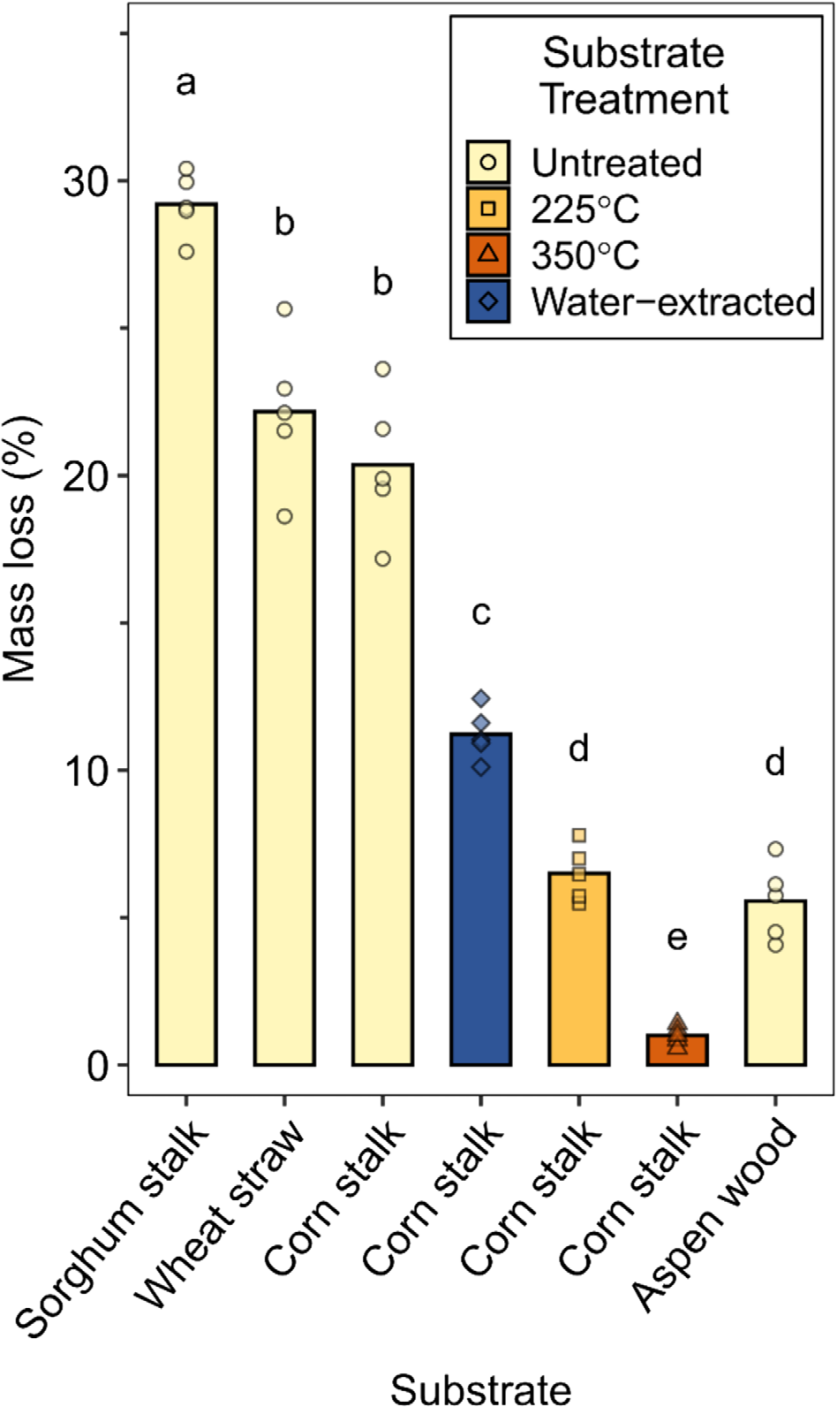
Mass loss of milled (< 20 mesh powder) lignocellulose substrates within nylon mesh bags after 5 weeks of decay by *Neurospora crassa* in soil‐filled jars. Bars and points indicate mass loss averages and mass loss of individual replicates (*n* = 5), respectively. Compact letter display indicates significant differences (*p* < 0.05) based on ANOVA and a post hoc Tukey HSD test. Corn stalk substrates, pre‐decay, were untreated, water‐extracted, heated at 225°C for 20 min, or heated at 350°C for 20 min. All other substrates, pre‐decay, were untreated.

*N. crassa* was capable of degrading all three grass species tested. As shown in Figure 4, sorghum stalk had the highest mass loss of 29.2% (±1.1 SD), whereas wheat and corn had slightly lower values (*p* < 0.05) of 22.2% (±2.5 SD) and 20.4% (±2.4 SD), respectively. Although significantly lower than untreated corn stalk (*p* < 0.05), mass loss for corn stalk pre‐extracted with water was substantial at 11.2% (±0.9 SD). Additionally, mass loss values for 225°C and 350°C‐treated corn stalk and aspen wood were 6.5% (±0.9 SD), 1.0% (±0.3 SD), and 5.6% (±1.3 SD), respectively, and were comparable to the values obtained in the first two experiments, despite the differences in culture setup and substrate preparation.

### 3.4 Chemical Characterisation of Corn Stalk Decay by *Neurospora Crassa*

**FIGURE 5:**
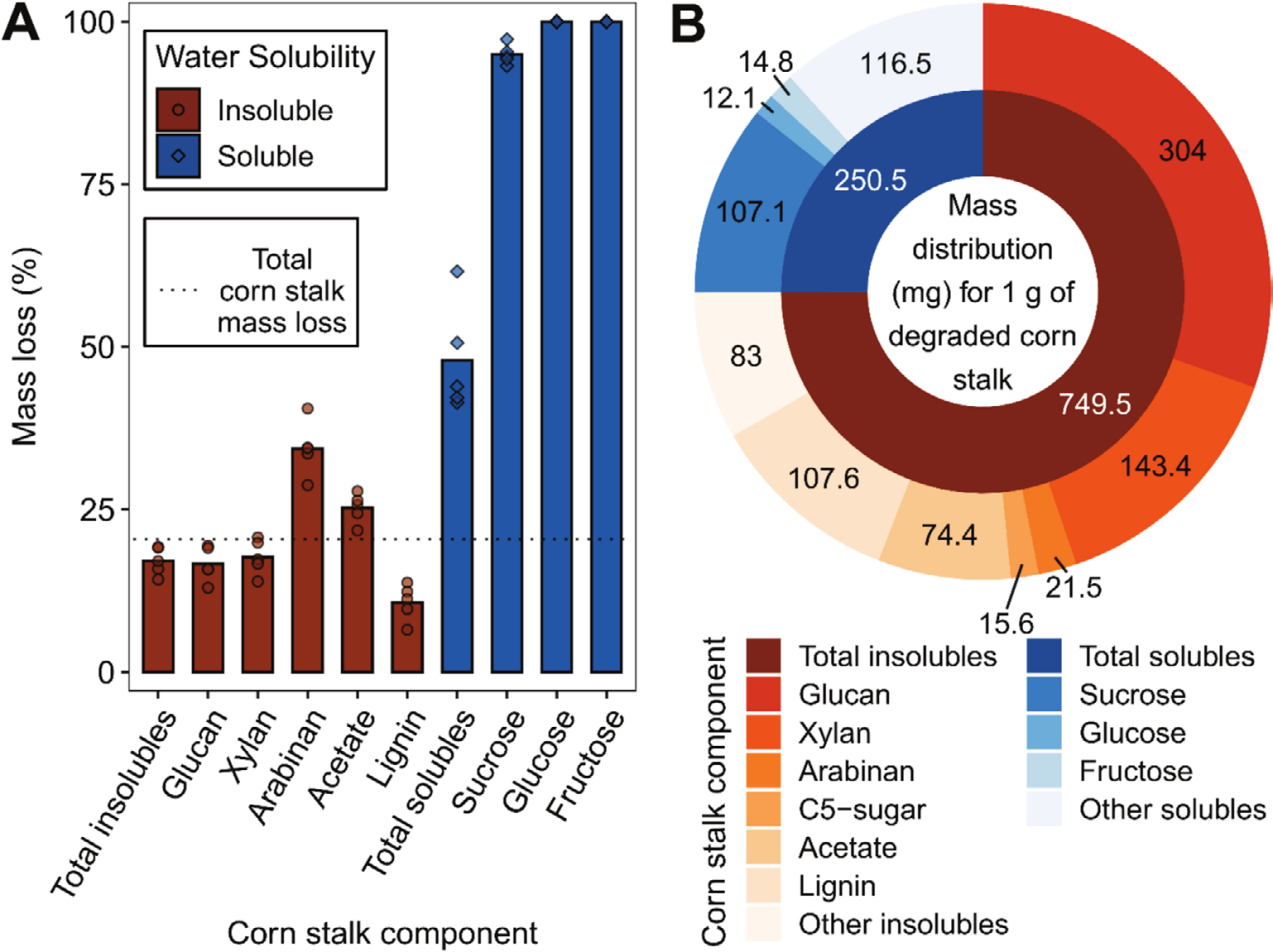
Mass loss of chemical components in untreated corn stalk (< 20 mesh powder) within nylon mesh bags after 5 weeks of decay by *Neurospora crassa* in soil‐filled jars. (A) Mass loss (%) from each component (bars and points represent averages and replicates (*n* = 5), respectively) in comparison to total corn stalk mass loss (dotted line). (B) Distribution of mass (mg) among components for 1 g of degraded corn stalk. Inner ring shows masses for total solubles and total insolubles, whereas outer ring shows masses for individual components within each of those categories.

Both water‐soluble (e.g., sucrose, fructose, glucose) and ‐insoluble (i.e., lignocellulose) compounds within untreated corn stalk were substantially degraded by *N. crassa* . As shown in Figure 5A, percent mass loss varied by compound, with glucose and fructose (water‐soluble) both having the highest mass loss of 100%, and lignin (water‐insoluble) having the lowest at 10.7% (±2.8 SD). When considering all water‐soluble compounds collectively, mass loss was 47.9% (±8.5 SD), which was significantly greater (*p* < 0.05) than total corn stalk mass loss (20.4% (± 2.4 SD)). Conversely, mass loss for water‐insoluble compounds, collectively, was significantly less (*p* < 0.05) than total corn stalk mass loss at a value of 17.1% (±2.2 SD). While *percent* mass loss for water‐soluble compounds was significantly greater than that for water‐insoluble compounds (*p* < 0.05), the *total* mass of degraded water‐insoluble compounds was significantly greater than that for soluble compounds (*p* < 0.05). This is demonstrated in Figure 5B, which shows that the mass, in milligrams, of water‐solubles and insolubles degraded per gram of total degraded corn stalk was 250.5 mg g^−1^ (±31.1 SD) and 749.5 mg g^−1^ (±31.1 SD), respectively. When considering the masses of specific compounds within these two fractions, values were highly variable, with glucan (water‐insoluble) having the largest mass of 304.0 mg g^−1^ (±20.1 SD) and glucose (water‐soluble) having the lowest at 12.1 mg g^−1^ (±4.1 SD).

## 4 Discussion

Despite the strong preference of *N. crassa* for burned substrates in post‐fire environments, the fungus showed no special capacity to degrade heat‐treated substrates (grass stalks or wood) relative to non‐fire‐associated fungi (*Gloeophyllum trabeum* or *Trametes versicolor*). This suggests that the natural proliferation of *N. crassa* on these substrates may be more strongly linked to fire‐resistant (e.g., heat tolerance; Shear and Dodge 1927; Lindegren 1932) and fire‐responsive (e.g., heat or furfural induced spore germination; Emerson 1948; Emerson 1954; Eilers and Sussman 1970; Pandit and Maheshwari 1996) traits rather than the fire‐adaptive trait of PyOM metabolism (Hopkins and Bennett 2024). *Neurospora* may survive the heat of a fire from within the substrate as an endophyte while competitors die (e.g., Kuo et al. 2014), or it may germinate in the soil and quickly colonise a freshly fire‐killed (i.e., plant immune defences eliminated) and potentially sterilised (i.e., endophytic fungi eliminated) substrate before competitors arrive (e.g., Pandit and Maheshwari 1996). In either case, *Neurospora* may avoid charred areas of the substrate and instead consume areas that remained relatively unmodified by fire. This is supported by the fact that *N. crassa* degraded untreated corn stalks and, to a lesser extent, stalks treated at 225°C (i.e., lightly pyrolyzed; some loss of hemicellulose) but not corn stalks treated at 350°C (i.e., heavily pyrolyzed; most hemicellulose lost, and some cellulose and lignin lost; substantial char formation). When exposed to fire naturally, the exterior of these stalks may be heavily charred, whereas the interior may only experience limited chemical modification after failing to reach sufficient temperatures for more complete pyrolysis. While this might explain the grass decay abilities we observed, it does not explain why *N. crassa* struggled to degrade wood substrates, regardless of heat treatment.

The enhanced ability of *N. crassa* to degrade grasses (corn stalk, sorghum stalk, wheat straw), relative to wood (aspen, spruce), is likely due to significant differences in substrate chemistry. For example, there are higher concentrations of water‐soluble sugars (e.g., sucrose) in grasses (e.g., > 6× higher, depending on species; Johnson et al. 2007; Giovannelli et al. 2011), and these sugars are easier to metabolise than lignocellulose polymers (e.g., cellulose, hemicellulose). This is supported by the fact that *N. crassa* almost completely degraded all water‐soluble sugars quantified (sucrose, glucose, fructose) for non‐heat‐treated corn stalk (Figure 5A), which explains roughly 13% of the total corn stalk mass degraded by the fungus (Figure 5B). However, enhanced degradation of grasses was not due solely to water‐soluble sugars, as evidenced by the fact that 75% of corn stalk mass loss was due to the degradation of water‐insoluble lignocellulose, while only 25% came from water‐soluble compounds, including sugars (Figure 5B). Additionally, even when water‐soluble compounds were extracted prior to decay, *N. crassa* was still able to degrade corn stalk (11% mass loss) better than wood (< 6% mass loss) (Figure 4). Combined, this suggests that *N. crassa* decay mechanisms may be better adapted to the chemistry of grass, rather than wood, lignocellulose (e.g., less total lignin, different lignin composition, different hemicellulose composition; Henriksson 2009; Scheller and Ulvskov 2010). We cannot rule out nitrogen, however, which is found in both the water‐soluble and ‐insoluble fractions, and is more concentrated in grasses (Johnson et al. 2007) than in wood (Cowling and Merrill 1966; Merrill and Cowling 1966).

One possible explanation for why *N. crassa* grows on burned trees in nature but struggles to degrade wood regardless of heat treatment severity or tree species is that *N. crassa* may be consuming the phloem and cambial layers after the tree has been killed by fire, when immune defences to fungal colonisation have been eliminated. Tree phloem and cambium are nutrient‐rich tissues under the bark and have a much higher nitrogen content and water‐soluble sugar content (e.g., potentially 100× and 9× higher, respectively, depending on species and sampling process; Cowling and Merrill 1966; Merrill and Cowling 1966; Giovannelli et al. 2011) than the woody tissue in the interior of the tree. While fires do often burn through the bark, phloem, and cambium layers, charring the wood beneath (e.g., fire scars from leeward vortices; Michaletz and Johnson 2007), it is common for much of the tree bark to remain intact even after the phloem and cambial layers have been heated to a lethal temperature (~60°C; Michaletz and Johnson 2007). In this scenario, the fungus could consume this nutrient‐rich and sterilised substrate without having to degrade any wood (charred or otherwise) or compete with endophytic fungi. This was also suggested by Jacobson et al. (2004) who found *Neurospora* proliferating exclusively under the bark of various tree species when studying post‐fire sites in western North America. While the primary mode of colonisation could not be confirmed, it was suggested that endophytic hyphae or conidia below the bark could survive a fire and then colonise the freshly killed phloem and cambium layers; however, there was no evidence at that time that *Neurospora* had an endophytic lifestyle. Since then, it has been suggested that *Neurospora* could live as a tree endophyte and survive heat treatment while within the substrate, as demonstrated for Scots pine ( *Pinus sylvestris* ) (Kuo et al. 2014). Population studies from regions other than western North America, however, have found *Neurospora* proliferating on the surface of charred bark, rather than beneath it (Jacobson et al. 2006). This suggests that the mechanism for *Neurospora* colonisation and proliferation on burned substrates may not be consistent across regions or substrate types; therefore, more comprehensive investigations are still needed to fully elucidate the relationship between *Neurospora* and charred tree substrates.

Our work yields novel insight into the relationship between *Neurospora crassa* and burned plant substrates, but there are some limitations to note. We tested only two heat treatment temperatures (225°C, 350°C), which limits the resolution of how heat treatment affects fungal decay. These temperatures were targeted for hemicellulose and cellulose degradation, respectively, but higher temperatures are needed for more advanced lignin degradation (Tillman et al. 1981; Rowell and Dietenberger 2012). Substrates treated at more extreme temperatures would, thus, have unique chemistry, and while we would not anticipate greater decay potential on these substrates within the current study design, they nonetheless remain untested. Additionally, the treatment temperatures we used affected the various substrate types differently (as judged by mass loss during heat treatment). This was especially true for 225°C, which resulted in ~11% mass loss for corn stalks but < 1% mass loss for both wood types (Figure S2). This may be due to the lower density of the corn stalk disks relative to the wood blocks (i.e., heats and pyrolyzes quicker) or the differences in substrate chemistry between wood and grasses (e.g., water‐soluble compounds, lignin, hemicellulose). It is challenging to heat‐treat different substrates ‘equally’, and testing a wider range of treatment temperatures could help make more accurate decay comparisons between substrates. It may also be useful to more thoroughly characterise the chemistry of different substrates before and after heat‐treatment and decay, to gain a more thorough understanding of how heat‐treatments modify each substrate and how these chemical modifications affect decay. Another limitation to our methodology was temporal resolution. While we did test three different time points for corn stalk decay by *N. crassa* we did not test multiple time points for wood, due to the comparatively low decay seen for both wood substrates after 5 weeks of growth. It is possible that, given more time, *N. crassa* could degrade wood more significantly than what we observed here, and the evaluation of decay at multiple time points could help clarify the maximum decay capacity for different wood substrates. Finally, the use of only five culture replicates in each of our microcosm experiments may have limited the statistical power of our mass loss comparisons. This may be especially true for the experiments utilising whole ‘block’ or ‘disc’ substrates (Figures 1 and 3) where mass loss variance was much greater than that for the experiment utilising milled (i.e., homogenised) substrates (Figure 4). Future work may, therefore, benefit from a higher replicate count to make cleaner mass loss comparisons between species and substrates.

While an analysis of *Neurospora* genetics was outside the scope of this work, future genetics work could yield additional insight into how this fungus interacts with PyOM. Comparative genomics and transcriptomics studies of fire‐associated fungi (Fischer et al. 2021; Steindorff et al. 2021, 2022) have yielded valuable insight into how these fungi interact with, and possibly metabolise, PyOM. Specifically, genes that are enriched within fire‐associated fungi (relative to non‐fire‐associated fungi) or upregulated on PyOM (relative to unmodified organic matter) have been identified and implicated in PyOM metabolism. Similar work with *Neurospora crassa* could help to clarify its genetic potential for PyOM metabolism. Results may corroborate our findings of poor PyOM degradation capacity or perhaps present a more complex picture for the ecology of *Neurospora* in post‐fire environments.

While much of the ecology of *N. crassa* in nature remains obscure, our work has provided some clarity into the saprotrophic capabilities of this fungus and its relationship with fire‐affected environments. We found that *N. crassa* was unable to significantly degrade charred plant biomass (grass or wood) and that it struggled to degrade wood regardless of heat treatment; however, it was able to degrade untreated grass substrates. This work not only enhances our overall understanding of *Neurospora* biology but may also benefit the field of fungal fire ecology, which is of growing interest and importance, as human activities continue to alter natural fire regimes globally (Fox et al. 2022).

## Author Contributions

**Hunter J. Simpson:** conceptualization, methodology, investigation, formal analysis, visualization, writing – original draft, writing – review and editing. **Jonathan S. Schilling:** funding acquisition, conceptualization, methodology, writing – review and editing.

## Conflicts of Interest

The authors declare no conflicts of interest.

## Supporting information


**Data S1.** Figures.

**Data S2.** Tables.

## Data Availability

Mass loss data for the agar‐block microcosm experiment can be found in Table S1. Mass loss data for the two soil‐block microcosm experiments, time series and litter bag, can be found in Tables S2 and S3, respectively. Corn stalk chemical component mass loss data is available in Table S4.
