# Supplementary material for: Saprotrophic Capabilities of Neurospora crassa on Charred Plant Biomass
Source: Environ Microbiol. 2025 Jun 24;27(6):e70132. doi: 10.1111/1462-2920.70132 (PMC12186714; doi:10.1111/1462-2920.70132)
Supplement: Supplementary file 1 — Data S1. Figures. [file EMI-27-e70132-s002.docx]

**Supporting Information**

**Title:** Saprotrophic capabilities of *Neurospora crassa* on charred plant biomass

**Authors:** Hunter J Simpson, Jonathan S Schilling

**
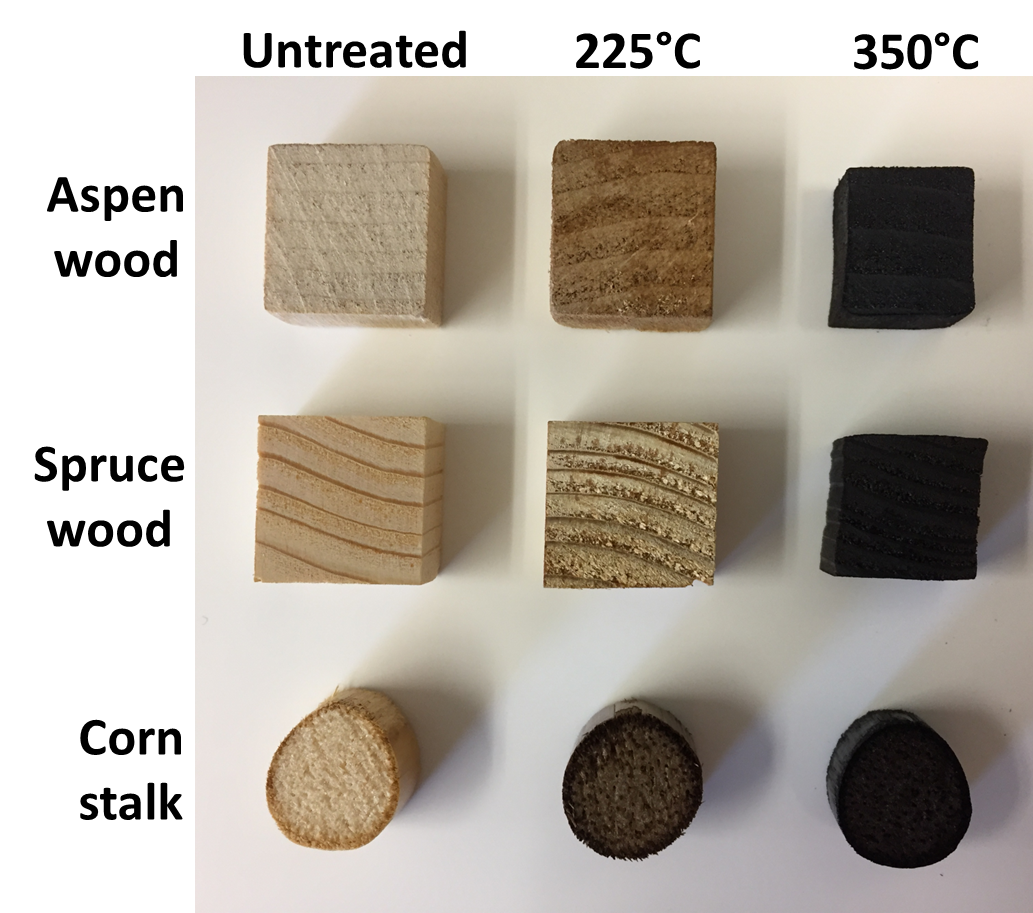
**

**Figure S1**. Visual comparison of substrates before and after heat treatment. Substrates include aspen and spruce sapwood blocks, as well as corn stalk internode disks. Heat treatments include 20 minutes of heating at 225˚C or 350˚C.

**
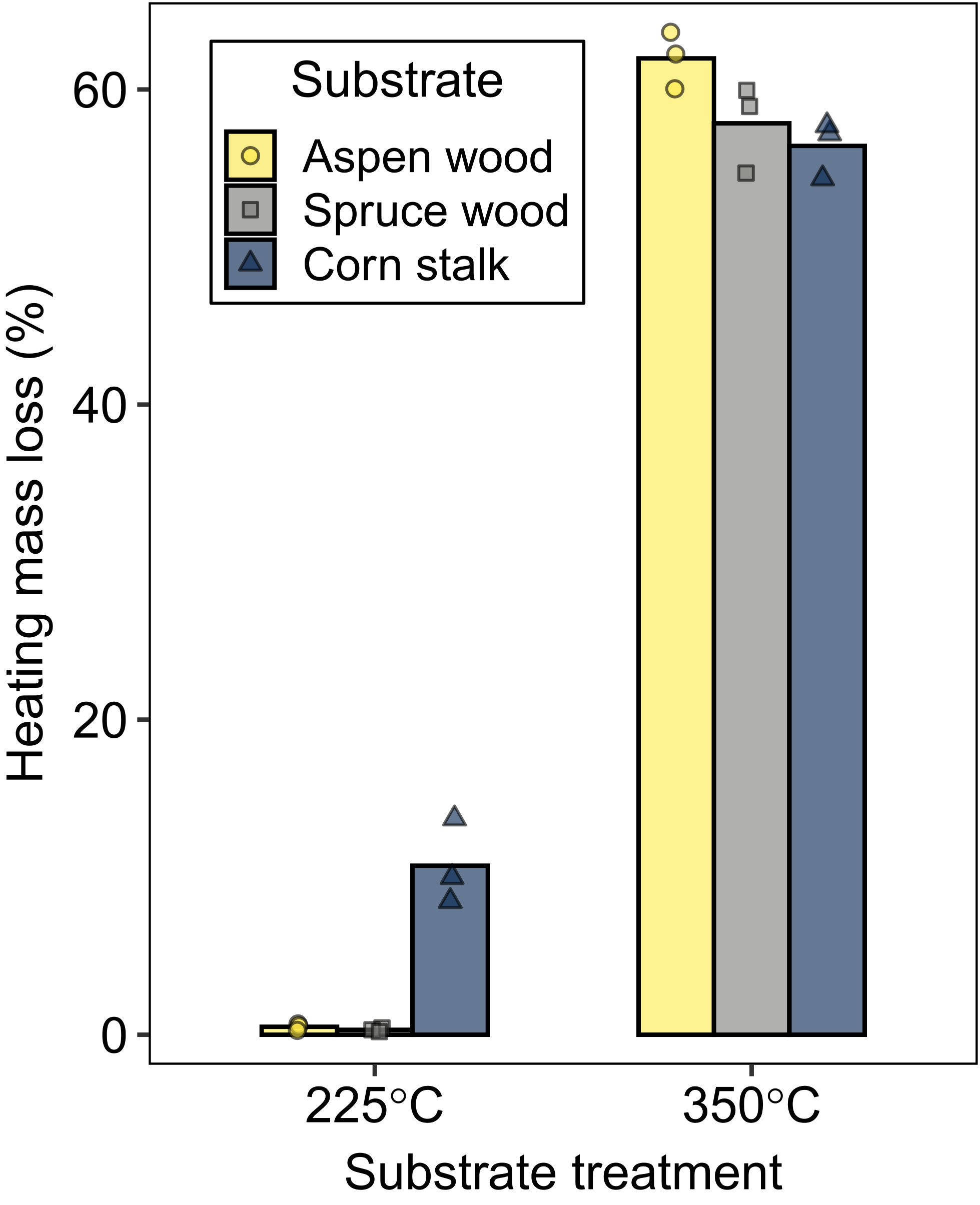
**

**Figure S2**. Mass loss of lignocellulose substrates due to heat treatment. Substrates include aspen and spruce sapwood blocks, as well as corn stalk internode disks. Heat treatments include 20 minutes of heating at 225˚C or 350˚C.

substrates before and after heat treatment. Substrates include aspen and spruce sapwood blocks, as well as corn stalk internode disks. Heat treatments include 20 minutes of heating at 225˚C or 350˚C.


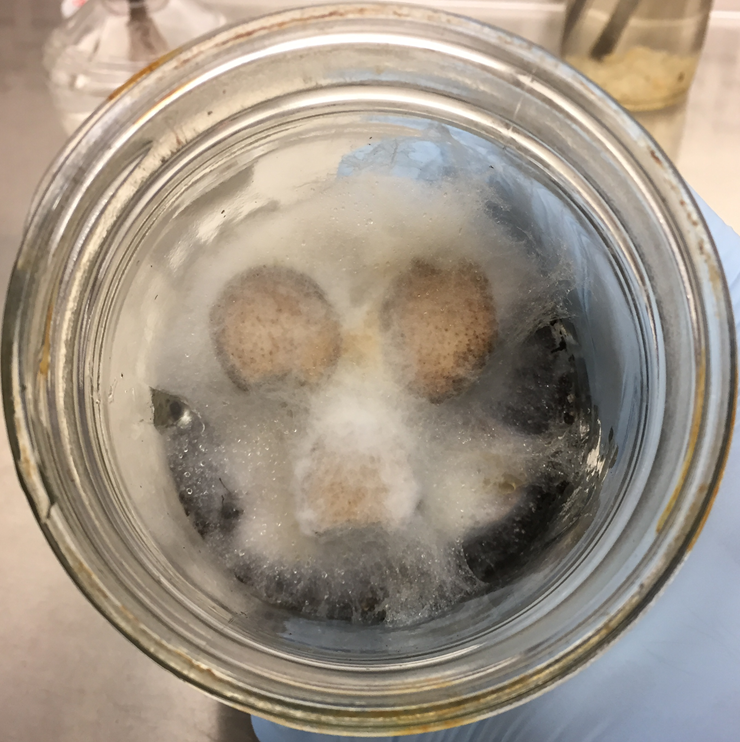


**Figure S3**. Soil-block jar microcosm with corn stalk disk substrates after 10 days of growth by *Neurospora crassa*. In this example, all three corn stalk disks are untreated and were harvested from the jar at three different time points: 10, 20, and 40 days.


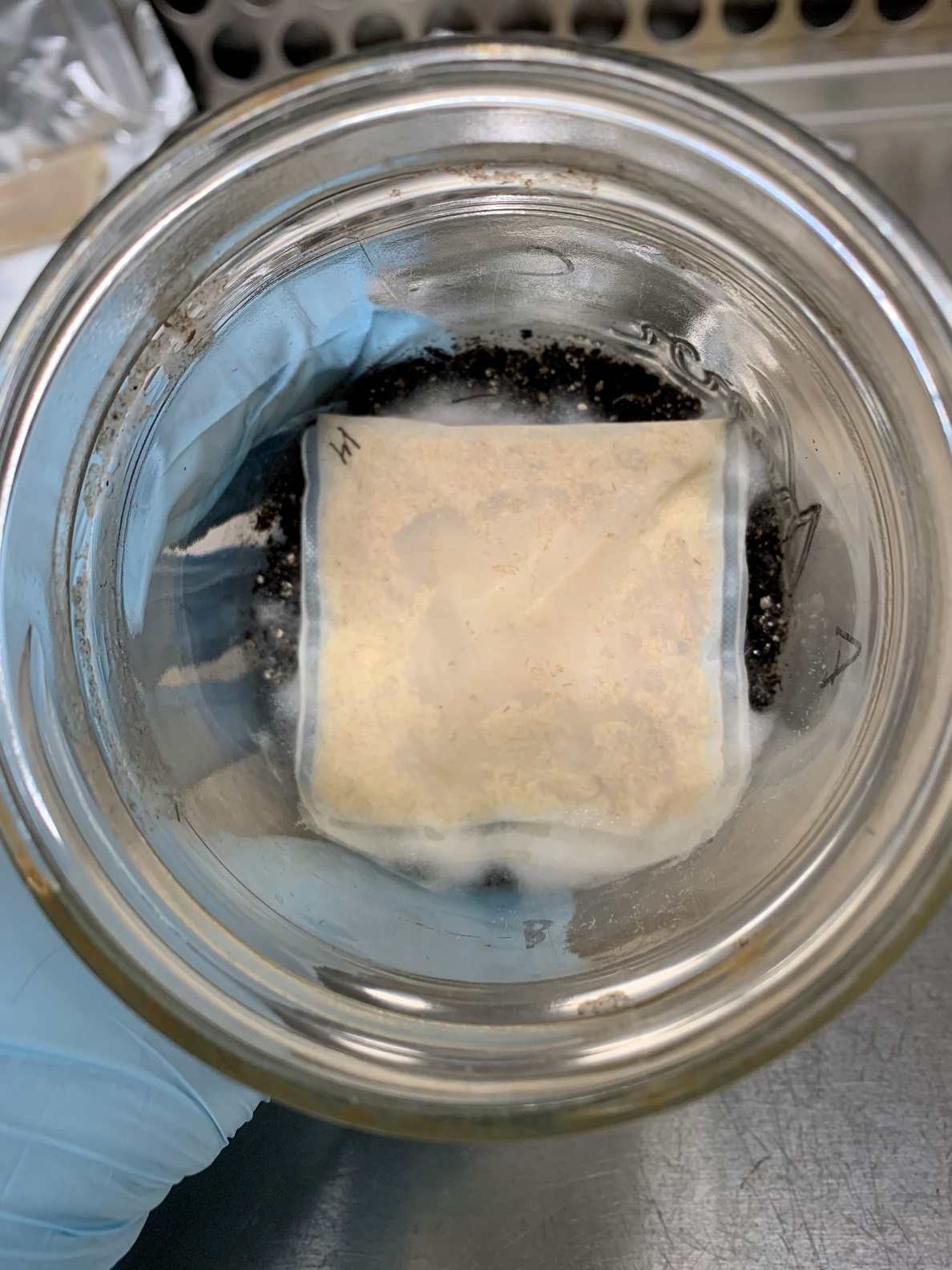


**Figure S4**. Soil-block jar microcosm with mesh bag of corn stalk powder (< 20 mesh) after five weeks of growth by *Neurospora crassa*.
